# Exploring Physical Activity Levels and Performance Among High-Intensity Transplant Athletes at the World Transplant Games

**DOI:** 10.1177/15269248251383949

**Published:** 2025-10-07

**Authors:** Bart Rienties, Leigh Martin, Fereshte Goshtasbpour

**Affiliations:** 1 Institute of Education Technology, 5488 The Open University, Milton Keynes, UK

**Keywords:** exercise outcomes, exercise physiology, research, quantitative methods, correlational, adherence, growth and development

## Abstract

**Introduction:**

While most transplant recipients remain sedentary posttransplant, some transplant recipients are able to meet and even exceed recommended physical activity levels.

**Research Question:**

The objective of this study was (1) to explore what physical activity levels 25 recipient athletes could sustain over a 7-month period while preparing for and participating in the World Transplant Games in cycling and/or triathlon, and (2) what intensity levels recipients sustained in competitive conditions.

**Design:**

The study adopted an observational descriptive research design and used physical activity self-reported data of 25 recipient athletes and self-tracked data from online social fitness network apps such as Strava. It also examined World Transplant Games performance metrics to evaluate transplant athletes’ capabilities in competitive settings.

**Results:**

Findings revealed that transplant athletes exceeded the current physical activity level guidelines by 300% over a 7-month period, with an average of 8.33 h per week of self-reported activities and 4.67 h per week of self-tracked moderate to intense physical activity.

**Conclusion:**

The findings highlighted the need to consider the capabilities of transplant athletes in reassessing the current physical activity guidelines since the results demonstrated that enhanced performance in competition settings was attainable through physical activity and support. There is a need for more personalised physical activity metrics and recommendations for transplant recipients.

## Introduction/Background

In recent years, the body of research into the effects of exercise and physical activity, in particular on health outcomes and the quality of life of transplant recipients, has grown.^1–3^ Physical activity can significantly contribute to posttransplant recovery and reduce the likelihood of developing chronic health conditions.^2,4^ Nonetheless, many recipients struggle to return to healthy norms of physical activity,^1^ in part due to a lack of health literacy, thereby increasing the possibility of developing secondary health conditions and reducing their health-related quality of life.^5^ Estimates of physical activity levels of recipients indicated that less than half of transplant recipients were sufficiently physically active.^1,2,6^

While there are some emerging physical activity guidelines for transplant recipients posttransplant,^1,7^ these guidelines are relatively generic and perhaps conservative. For example, in a systematic review of physical activity guidelines for kidney transplant recipients, Baker et al^1^^(p.3)^ indicated that, “We recommend [kidney transplant recipients] aim for 150 min of moderate to vigorous physical activity a week (or 75 min vigorous physical activity) as per the UK Chief Medical Officers’ Guideline.” Medical professionals often lack guidance and advice on appropriate ways to encourage suitable physical activity,^8^ and transplant recipients often struggle to interpret these guidelines.^9^ In particular, which types of sports and physical activity might be appropriate for a transplant recipient and how to safely build up to a sustainable level of physical activity over time has received limited empirical attention.^9^ Furthermore, there are limited empirical studies that report on exercise using objective data,^10,11^ specifically when measured over a substantial period of time in real-world settings.^3,12^

## Specific Aim

The first aim of this study was to describe and compare self-reported and objective exercise data from transplant athletes to explore the level of physical activity transplant athletes can sustain over a 7-month period while preparing for and participating in the World Transplant Games (WTG) in cycling and/or triathlon. The second aim was to describe the intensity levels (eg, heart rate and power/best average speed) that transplant athletes achieved during actual competitive events.

## Design/Methods

### Design

This was an observational descriptive research study of transplant recipients. This study was approved by the local institutional review board (HREC/4787). Informed consent was obtained from all individuals included in the study.

### Setting

Participants of the 2023 WTG in Perth, Australia, were invited to join an online interview via Microsoft Teams in the period July–August 2023. The interviews occurred via the participants’ personal computer at the time and place most comfortable for recipients, around 3 months after the Transplant Games.

### Population

The target population was adults who received a transplant and participated in cycling and/or triathlon in Perth. Participants had to have a stable function and a letter of support from their medical consultant to be allowed to participate in the WTG. Of all the participants of the Transplant Games, 121 recipients participated in at least 1 of the triathlon and/or cycling events, with an average age of 44.71 (SD 13.36), 30 (25%) females and 91 (75%) males, 102 participated in cycling, 44 in triathlon, and 25 did both. Participants were from Australia (*N* = 29), UK (*N* = 16), Belgium (*N* = 9), France (*N* = 9), USA (*N* = 8), Italy (*N* = 5), Brazil (*N* = 5), South Africa (*N* = 4), Switzerland (*N* = 4), Uruguay (*N* = 4), and 17 other countries with 3 or less participants. Race performance data (ie, time, best average speed, and finish position) were gathered during the WTG on all 121 transplant participants, and these data were publicly available.

Publicly available data from the Strava application was used to have a nontransplant population comparison group. No demographic data were available for the nontransplant population.

### Sampling

Potential recipients to be interviewed were recruited in 2 ways based on their gender, age group, country, and participation in cycling and/or sprint triathlon. The research team approached 28 participants directly in July 2023, and 22 (79% response rate) were subsequently interviewed. Second, via social media, including the official WTG website and several Strava and Facebook groups (eg, Transplant Cyclists of the World), an open invitation was posted for any eligible participant, whereby 5 additional participants were recruited, leading to 27 participants. Participants had to be able to speak English, German, or Dutch (languages spoken by the research team) to join the interview.

Of the 121 transplant participants, 46 uploaded their Strava data (eg, heart rate, power, and best average speed) on the closed-circuit racetrack, and data were used.

To have a comparison group, data were used on 144 nontransplant athletes who recorded in the Strava app at least 1 lap on the same closed-circuit racetrack during the period 2014–2023.

### Data Collection

#### Online Interviews on Physical Activity

Interviews included 16 semistructured questions and lasted between 45 and 60 min, following the interview approach used in previous studies^6,9^ in 4 overlapping parts (eg, what is physical activity; why participants joined the transplant games; and participants’ physical activity levels and type of transplant). Interviews were conducted online using MS Teams, whereby audio was automatically transcribed (with explicit permission from participants). These transcripts were checked, cleaned, and subsequently sent back to participants for sense checking and final agreement. Participants were asked about their physical activity (hours/week) before receiving their transplant and physical activity (hours/week) in 2023.

#### Physical Activity Data From Online Social Fitness Network Apps

After the online interview had taken place and with explicit permission from participants, objective physical activity data using global positioning system (GPS) were collected using common cycling and triathlon online social fitness network apps,^12–14^ such as Strava, Garmin, etc. In Strava, aggregated statistics per participant were extracted on 8 August 2023 for the number of cycling, running, and swimming activities, distance (in kilometres), and total time completed for these activities. The recorded average training load per week from 1 January 2023 to 1 August 2023 was chosen because it would capture 15 weeks of physical activity before the WTG in the third week of April 2023, participating in those games, and subsequently 14 weeks of physical activity afterwards (ie, measuring whether participants remained active afterwards). For consistency, participants were asked about their app habits (ie, did they share all their activities, or uploaded only a selection of activities). If particular cycling, running, and/or swimming activities were not publicly available, respective participants were asked to add their data manually to an anonymous spreadsheet and included in the objective physical activity analysis. The combination of these 3 activities was used as a proxy for recorded exercise time per week. If participants did not use a GPS device, they were excluded from the objective physical activity analysis.

#### Performance Data From WTG

The WTG race performance data were gathered from the results of the cycling and sprint triathlon races from the official website.^15^ As these races were conducted with official timing chips and raced under competitive conditions on closed race circuits, these activities represented the athletes’ maximum (near) performance. These publicly available data contained the age groups (age range: 18–29, 30–39, 40–49, 50–59, and 60–69) and gender in which transplant athletes competed, their average speed, and their absolute position. Participants could compete in up to 3 cycling races (ie, 10K individual time trial, 30K race, and 20K triathlon), and therefore, the main indicator for performance was the best average speed, which was computed as the highest recorded average speed by a participant during these races. The best average speed and heart rate data were abstracted for the nontransplant athletes for a comparison group.

### Data Analysis

All data were analyzed with SPSS v29 (IBM Statistics Armonk, NY, USA). Quantitative data from the interviews were used on participants’ pre- and posttransplant physical activity in hours per week and their type of transplant. Self-reported pretransplant and posttransplant data, as well as running and swimming activities, were not normally distributed, and therefore, nonparametric tests were used. Recorded exercise time per week, cycling activities, and best average speed were normally distributed, and analysis of variances were used to compare across transplant types. Pearson correlations were used to test the correlations between self-reported and objective data.

## Results

Of the 121 transplant recipients who participated in triathlon or cycling at the WTG, 27 participants consented and were interviewed, and 46 uploaded transplant game preparation exercise data in Strava (including 25 out of 27 interviewed participants). All transplant game event data were used.

Four (14%) of the interviewed participants identified as female, and 23 (86%) identified as male, and the average age was 54.9 years. 96% participated in cycling, 37% participated in a sprint triathlon, and 33% participated in both disciplines. The 27 participants included 9 bone marrow, 9 kidney, 4 liver, and 3 heart transplant recipients (Table 1).

**Table 1.** Characteristics of Transplant Recipients Who Participated in Cycling or Triathlon Events (*N* = 27).

Characteristics	*N* (%)	
Sex, male	23 (85%)	
*Age group*		
18–29	3 (11%)	
30–39	4 (15%)	
40–49	5 (18%)	
50–59	10 (37%)	
60–69	5 (18%)	
*Transplant type*		
Bone marrow	9 (33%)	
Heart	3 (11%)	
Kidney	9 (33%)	
Liver	6 (22%)	
*Continent of birth*		
Africa	1 (4%)	
Australasia	6 (22%)	
Europe	18 (66%)	
North America	2 (7%)	*Mean (SD)*
*Time since transplant*		12.25 (6.96)
*Physical activity prior to transplantation, hours/week*		5.44 (4.23)
*Physical activity during 2023 (prior to transplant games), hours/week*		8.33 (3.09)

There were 94 who were not consented into the study, but the race performance data that were publicly available were collected. No statistically significant differences were found for gender and age between the 27 recipients who participated in the interview and the 94 recipients who raced in Perth but did not join the interview.

Those who participated in the interview on average had a higher best average speed (*M*_27_ = 32.90, SD_27_ = 4.68; *M*_94_ = 27.92, SD_94_ = 4.54, *F* = 24.810, *P* < .001, *η*^2^ = .173), and the percentage of participants winning a medal was higher (*M*_27_ = 62%, *M*_94_ = 40%, *F* = 4.382, *P* < .05, *η*^2^ = .036) relative to those who did not join the interview.

### Self-Reported Physical Activity Levels Before Transplant and in the First 7 Months of 2023

To address the first research aim of what physical activity levels transplant athletes can sustain over a 7-month period while preparing for and participating in the WTG in cycling and/or triathlon, participants’ (self-reported) physical activity before their transplant and their self-reported physical activity in 2023 from the interviews were analyzed. Before participants became ill, on average, they spent 5.44 h per week (pw) (SD = 4.23, range 1–11) on physical activity. Eight participants had limited physical activity before transplant (<2 h pw), while 8 participants exercised extensively (>8 h pw). Fourteen (52%) participants increased their self-reported physical activity over time in a typical week in 2023, whereby on average 8.33 h pw (SD = 3.09, range 1–11) was spent on physical activity. On average, physical activity duration increased by 2.89 h per week after transplant, indicating that 26 out of 27 transplant athletes reached similar or even higher (self-reported) physical activity duration after transplant.

### Objective Physical Activity Levels During the 7 Months of 2023

Recorded objective data from online social fitness network apps during the first 7 months of 2023 indicated that, on average, participants recorded 4.67 h per week in moderate to vigorous intensity physical activity in cycling, running, and/or swimming (SD = 2.67, range: 1.44–8.48). No significant differences were found for the number of recorded activities and distance by type of transplant (Table 2). Substantial training kilometers were obtained in these 7 months, averaging 95.47 km (SD = 55.23, range = 11.47–250.57) per week. Except for 1 person, the recorded data in terms of hours per week were lower than the self-reported physical activity data collected during interviews. There was a moderately strong correlation between self-reported physical activity duration in 2023 and recorded exercise per week (rho = .435, *P* < .05), indicating that participants could reasonably accurately recall their physical activity duration.

**Table 2.** Self-Reported and Objective Physical Activity Data for 7 Months in 2023 Per Week by Transplant Type.

Activity	Bone marrow (N = 9)	Heart (*N* = 3)	Kidney (*N* = 9)	Liver (*N* = 4)		Kruskal – Wallis	
	*M* (SD)	*M* (SD)	*M* (SD)	*M* (SD)	*F*-value		*P*-values
Physical activity in 2023 (self-reported) (hours/week)	9.50 (1.77)	10.33 (1.15)	7.89 (2.85)	7.00 (4.00)		3.122	.373
Number of recorded exercises in the App	4.99 (2.23)	6.75 (1.99)	3.87 (1.60)	4.34 (2.59)	1.574		.225
Number of cycling activities	3.83 (2.25)	4.53 (1.43)	3.09 (1.13)	2.01 (0.45)	2.147		.125
Number of running activities	0.69 (1.42)	0.56 (0.96)	0.62 (0.97)	1.02 (1.17)		1.161	.762
Number of swimming activities	0.31 (0.43)	0.00 (0.00)	0.19 (0.38)	0.59 (0.87)		3.442	.328
Cycling distance (km)	108.66 (71.75)	147.99 (36.16)	78.30 (44.54)	73.77 (31.68)	1.755		.187
Running distance (km)	5.66 (12.53	6.14 (10.64)	3.64 (5.64)	9.11 (10.97)		1.392	.708
Swimming distance (km)	0.30 (0.42)	0.99 (1.71)	0.18 (0.36)	1.25 (1.78)		2.383	.497

### Performance and Intensity During Competitive Events

**Figure 1.**
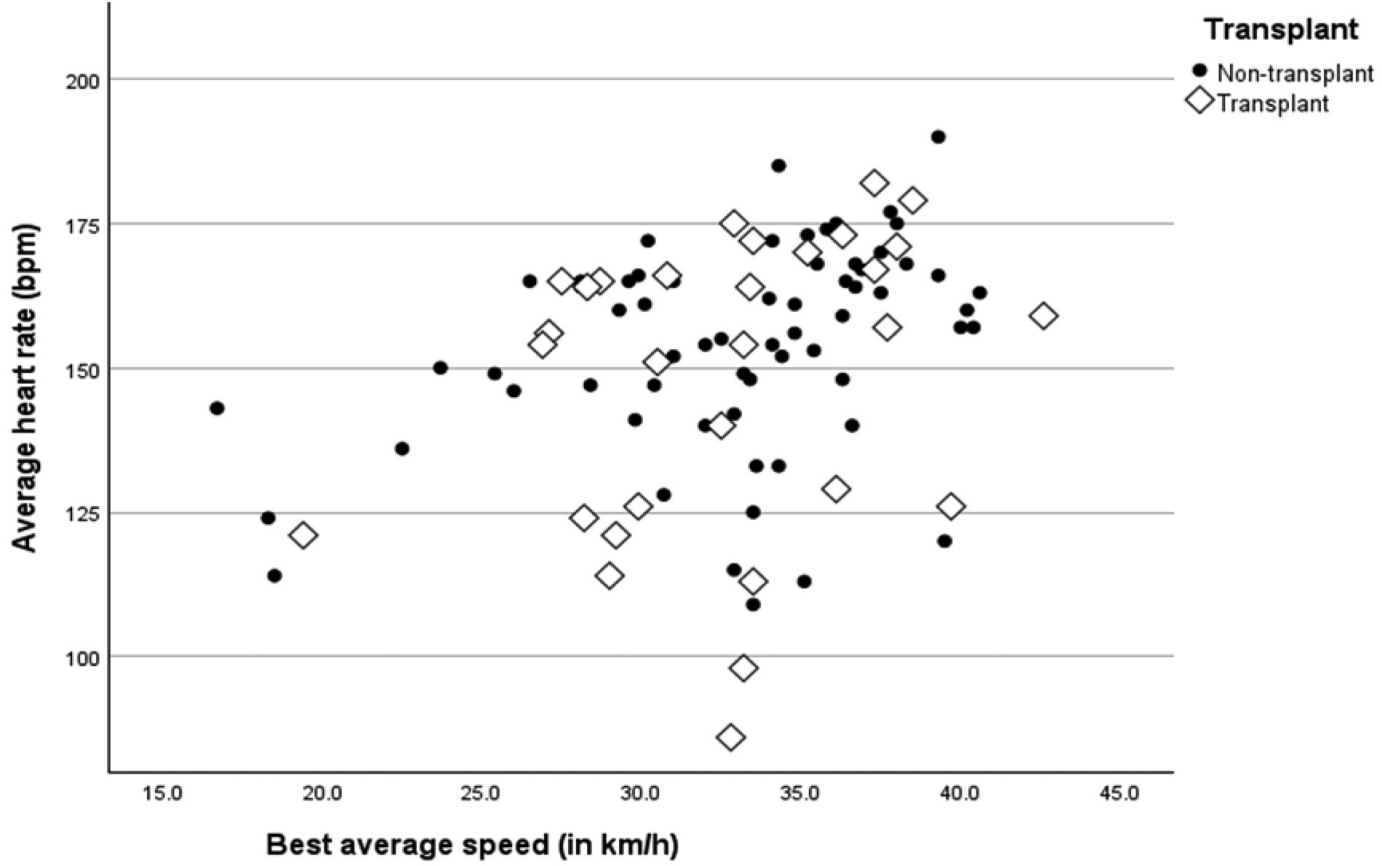
Best average speed on the World Transplant Games raceway and average heart rate. *Note*: Comparison of publicly accessed heart rate data from Strava for 30 transplant recipients and 65 nontransplant public members who used the same racetrack.

When comparing public data available on Strava of 46 transplant participants on the WTG racetrack of their best average speed (in km/h) for 1 lap (*M* = 32.23, SD = 5.38) with 144 nontransplant cyclists (*M* = 32.40, SD = 4.88), no significant differences were observed (*F* = .040, *P* = .841). Similarly, the average heart rate of 30 recipients who made their heart rate data available on Strava (*M* = 148, SD = 25.82) showed no significant difference was found with the 65 nontransplant cyclists who also shared their heart rate data (*M* = 153.35, SD = 18.0, *F* = 1.563, *P* = .214), see Figure 1.

There were several significant correlations between the best average speed and their physical activity programme in the first 7 months of 2023, which are displayed in Table 3. Self-reported physical activity before transplant in hours and self-reported physical activity in 2023 were not significantly correlated with the best average speed. In contrast, recorded exercise in online social fitness network apps was strongly positively correlated with the best average speed (rho = .640, *P* < .01). This indicated that those transplant athletes who were more physically active posttransplant were able to complete these races with a higher speed, but only objectively recorded exercise was a predictor for sport performance. Subsequent analyses per physical activity indicated that recorded cycling distance covered per week (rho = .647, *P* < .01) and number of cycling activities per week (rho = .435, *P* < .05) were correlated with the best average speed, but none of the running or swimming proxies. Perhaps surprisingly, age did not significantly correlate with any physical activity metrics or race performance metrics, indicating that age was not a barrier for those high-intensity transplant athletes competing in cycling and/or triathlon. On average, female participants had a lower best average speed (rho = −.547, *P* < .01) and reported lower physical activity in 2023 relative to male participants.

**Table 3.** Correlation Matrix Between Performance, Physical Activity Duration, and Demographics.

	*M*	SD	1	2	3	4	5
1. Best average speed (km/h)	33.34	4.47					
2. Physical activity before transplant (self-reported in hours)	5.40	4.16	.305				
3. Physical activity in 2023 (self-reported)	8.52	2.79	.340	.434*			
4. Recorded exercise in app (in hours)	4.67	2.14	.640**	.172	.435*		
5. Age	44.40	12.90	−.002	.186	.357	.004	
6. Sex (1 = male, 2 = female)	1.08	0.28	−.547**	−.173	−.380	−.222	−.547**

*N* = 25, *P < 0.5, **P < .01.

## Discussion

In most of the medical literature on physical activity for transplant recipients, there seems to be consensus that most transplant recipients were less active posttransplant, and most transplant recipients did not meet physical activity guidelines.^1,2,8^ This study focused on a specific group of high-intensity transplant athletes engaged in cycling and triathlon at the 2023 WTG. By integrating both self-reported and objective physical activity and intensity data from online social fitness network apps such as Strava,^12–14^ a deeper understanding of the physical activity levels maintained by transplant athletes during a 7-month period was established, roughly 3 months before and 3 months after the games. The results provided important considerations for both healthcare professionals and transplant recipients to understand and promote posttransplant physical activity.

One of the main findings of this study was that the physical activity levels achieved and sustained by high-intensity transplant athletes were higher than general physical activity guidelines for transplant recipients. On average, participants reported engaging in 8.33 h of physical activity per week, which was 3 times higher than the 150 min per week of moderate to vigorous physical activity recommended by UK guidelines^1,7^ and substantially more than reported in other transplant studies.^1,2,6^ Except for 1 participant, all other transplant athletes were equally or more active posttransplant, in contrast to previous literature.^1–3^

Objective data demonstrated that these transplant athletes engaged in an average of 4.67 h per week of moderate to vigorous physical activity in terms of cycling, running, and/or swimming over a sustained period of time, which was 3 to 4 times more than current physical activity guidelines.^1,7^ While the participants who joined the interview had similar demographic characteristics and similar recorded training activities as the other transplant recipients who did not participate in the interview, perhaps it is not unexpected that relatively faster and more medal-winning participants joined the interviews as they were keen to share their success stories.

As a second aim, this study sought to explore the intensity levels exhibited by transplant athletes during competitive events of cycling and/or triathlon. The analysis of performance data revealed that many transplant athletes competed at intensity levels of speed and heart rate comparable to nontransplant athletes. Additionally, there were no significant differences in average speeds or heart rates between transplant athletes and nontransplant cyclists on the closed racetrack, suggesting that transplant recipients can achieve relatively high-intensity levels of physical performance.

No correlations were found for athletic performance under competitive race conditions (ie, best average speed), self-reported pretransplant physical activity levels, and self-reported activity levels in 2023. In contrast, objective data and specifically cycling data (ie, distance per week and number of cycling activities per week) were positively correlated with performance. This aligns with a study that found few correlations between self-reported and objective physical activity levels among adults.^12^ Another study found that objective physical activity hours per week were substantially lower than self-reported physical activity levels.^10^ This might indicate that participants might overestimate their physical activity levels before and after the WTG, and therefore, collecting objective physical activity data would provide an accurate measurement of transplant participants' physical activity levels.

This study was not without its limitations. It was limited by a relatively distinct sample that consisted of highly active transplant athletes, who were not representative of the broader transplant population. This study specifically chose these highly active transplant athletes to provide a reference of what could be achieved among some transplant recipients. The use of self-reported data may introduce bias,^10^ although the use of objective online social fitness network data helped to mitigate these concerns.^12^

## Conclusion

This study provided valuable insights into the physical activity behaviors and capabilities of high-intensity transplant athletes. By challenging existing physical activity guidelines and advocating for more personalized approaches, one hopes to promote better health outcomes for all transplant recipients. In line with common physical activity literature,^10,11^ the data suggested a common link between physical activity duration, intensity, and performance among transplant athletes. Future studies could benefit from exploring the long-term health outcomes of different levels and intensities of physical activity among transplant recipients and consider a larger and more diverse sample to validate these findings.

These findings challenge some of the conservative recommendations of current physical activity guidelines for transplant recipients, which may not adequately consider the potential for high-intensity activity, and might discourage some transplant recipients from stretching their physical activity levels to or even beyond 150 min. An important implication of this study is the need for more personalized physical activity guidelines that take into account the diverse capabilities and experiences of transplant recipients. Medical professionals should consider tailoring recommendations to align with the individual's goals and fitness levels, encouraging those who have the physical capacity to engage in higher-intensity activities.
